# Comparison of nivolumab and sorafenib for first systemic therapy in patients with hepatocellular carcinoma and Child‐Pugh B cirrhosis

**DOI:** 10.1002/cam4.4906

**Published:** 2022-06-02

**Authors:** William J. Chapin, Wei‐Ting Hwang, Thomas B. Karasic, Anne Marie McCarthy, David E. Kaplan

**Affiliations:** ^1^ Division of Hematology‐Oncology, Department of Medicine, Perelman School of Medicine University of Pennsylvania Philadelphia Pennsylvania USA; ^2^ Department of Biostatistics, Epidemiology and Informatics University of Pennsylvania Philadelphia Pennsylvania USA; ^3^ Division of Gastroenterology and Hepatology, Department of Medicine, Perelman School of Medicine University of Pennsylvania Philadelphia Pennsylvania USA; ^4^ Division of Gastroenterology and Hepatology, Department of Medicine Corporal Michael J. Crescenz VA Medical Center Philadelphia Pennsylvania USA

**Keywords:** comparative effectiveness research, hepatocellular carcinoma, liver cirrhosis, nivolumab, propensity score, proportional hazards models, sorafenib

## Abstract

**Background:**

Patients with decompensated cirrhosis are excluded or underrepresented in clinical trials of systemic therapies for hepatocellular carcinoma (HCC) and comparisons of available therapies are lacking. We aimed to compare overall survival for patients with HCC and Child‐Pugh B cirrhosis treated with nivolumab or sorafenib as first systemic treatment.

**Methods:**

We performed a retrospective cohort study in patients with HCC and Child‐Pugh B cirrhosis treated at Veterans Affairs medical centers to compare overall survival, adverse events, and reason for discontinuation of therapy between patients treated with nivolumab or sorafenib as first systemic treatment. All statistical tests were 2‐sided.

**Results:**

Of those meeting inclusion criteria, 431 patients were treated with sorafenib and 79 with nivolumab. Median OS was 4.0 months (95% CI 3.5–4.8) in the sorafenib cohort and 5.0 months (95% CI 3.3–6.8) in the nivolumab cohort. In the multivariable Cox proportional hazards model, nivolumab was associated with a significantly reduced hazard of death compared to sorafenib (HR 0.69; 95% CI 0.52–0.91; *p* = 0.008). In a secondary analysis using propensity score methods, results did not reach statistical significance (HR 0.77; 95% CI 0.55–1.06; *p* = 0.11). Treatment was discontinued due to toxicity in 12% of patients receiving nivolumab compared to 36% receiving sorafenib (*p* = 0.001).

**Conclusion:**

In patients with HCC and Child‐Pugh B cirrhosis, nivolumab treatment may be associated with improved overall survival and improved tolerability compared to sorafenib and should be considered for the first systemic treatment in this population.

## Graphical abstract

Patients with hepatocellular carcinoma (HCC) and Child‐Pugh B cirrhosis have poor prognosis and no comparative data exists for guideline‐directed first line systemic therapies in this patient population. In this retrospective cohort study of patients with HCC and Child Pugh B cirrhosis treated at VA medical centers, nivolumab treatment was associated with improved overall survival in multivariable analysis and less treatment discontinuation for toxicity compared to sorafenib. Nivolumab should be considered as a first systemic therapy option in this patient population.

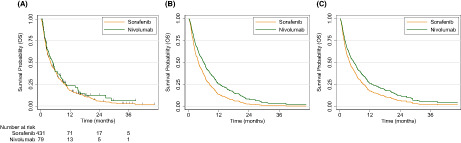

## 1 INTRODUCTION

Up to 90% of hepatocellular carcinoma (HCC) occurs in patients with cirrhosis and prognosis depends not only on anatomic staging and biological features of the cancer, but also on liver function.^1^ Liver function is typically assessed with Child‐Pugh classification ranging from Child‐Pugh A, representing compensated cirrhosis, to Child‐Pugh B and C, representing decompensated cirrhosis.^2^ The initial systemic therapy choice for most patients with Child‐Pugh A cirrhosis is the combination of atezolizumab plus bevacizumab, which demonstrated improved overall survival (OS) compared to the prior standard of care, sorafenib, in the IMbrave‐150 phase III randomized trial.^3^ However, this and other trials of systemic therapy have excluded or enrolled limited numbers of patients with Child‐Pugh B cirrhosis, as in this population complications of liver dysfunction are a competing cause of mortality compared to progressive HCC.^3^, ^4^, ^5^, ^6^ For this reason administering systemic therapy in patients with Child‐Pugh B cirrhosis is associated with increased risks and lower potential benefits.

There is limited prospective data establishing the efficacy and safety of sorafenib and nivolumab as options for first systemic therapy in patients with Child‐Pugh B cirrhosis. The use of sorafenib in patients with Child‐Pugh B cirrhosis is supported by results from the GIDEON registry, a real‐world, prospective registry examining safety and efficacy outcomes for patients receiving sorafenib for hepatocellular carcinoma in clinical practice that included 666 patients with Child‐Pugh B cirrhosis. The study observed a median OS of 5.2 months (6.2 months in patients with Child‐Pugh B7 cirrhosis, 4.8 months in patients with B8 cirrhosis, and 3.7 months in patients with B9 cirrhosis), a rate of discontinuation due to adverse events of 40% among Child‐Pugh B patients, and a similar toxicity profile compared to patients with Child‐Pugh A cirrhosis treated with sorafenib.^7^ Nivolumab was evaluated in patients with Child‐Pugh B cirrhosis in CheckMate‐040, a prospective, non‐comparative clinical trial.^8^ Among 49 patients with Child‐Pugh B cirrhosis (76% with B7 cirrhosis and 22% with B8 cirrhosis), median OS was 7.6 months and 4% of patients discontinued treatment due to adverse events with an overall toxicity profile similar to that of patients with Child‐Pugh A cirrhosis treated with nivolumab.^8^ CheckMate‐459 provides the highest quality comparative data of sorafenib to nivolumab to date, but only included patients with Child‐Pugh A cirrhosis. It found a trend towards improved OS that was not statistically significant (HR 0.85; *p* = 0.075) and a higher objective response rate (15% vs. 7%) for nivolumab compared to sorafenib.^5^ Additionally, nivolumab was associated with improved health‐related quality of life compared to sorafenib.^9^

While sorafenib and nivolumab have prospective data supporting their use in patients with Child‐Pugh B cirrhosis, there are no head‐to‐head comparisons of these treatment options in this patient population. We aimed to compare OS of patients with HCC and Child‐Pugh B cirrhosis treated with sorafenib or nivolumab in a real‐world setting and hypothesized that the use of nivolumab would be associated with longer OS compared to sorafenib. Additionally, we aimed to compare the incidence rates of key adverse events on treatment and the reasons for discontinuation of therapy.

## 2 METHODS

### 2.1 Study design and participants

We performed a retrospective cohort study of patients with HCC and Child‐Pugh B cirrhosis undergoing first systemic treatment using electronic health record and claims data from the Veteran Affairs administration Corporate Data Warehouse. Ethical approval was sought and the study was deemed exempt by the IRB at the Corporal Michael J. Crescenz Veterans Affairs Medical Center in Philadelphia, PA.

Patients were included if they had a diagnosis of HCC, underwent first systemic treatment with sorafenib or nivolumab between September 22, 2017, the date of FDA accelerated approval for nivolumab in the second‐line setting in patients with HCC, and September 11, 2021, and had Child‐Pugh B cirrhosis at the time for first systemic treatment.^10^ Child‐Pugh score and class were determined by a previously validated algorithm, though the score was adjusted by subtracting points for INR elevation for patients on systemic anticoagulation.^11^ Patients with a history of liver or organ transplant, patients who were found to have received prior systemic therapy, or patients who were being treated for a different malignancy with no evidence of active HCC were excluded.

### 2.2 Exposure, outcomes, and covariates

The primary exposure of interest was sorafenib or nivolumab use as first systemic treatment and the primary outcome was OS from time of first systemic treatment until death, or censoring for the time of last follow up in the VA system or November 15, 2021, whichever occurred first. To avoid misclassification of treatment exposure, all clinical charts were reviewed to ensure the validity of treatment classification and date of first treatment, that patient had not received prior systemic treatment, and that systemic treatment was being used for active HCC rather than another malignancy. Covariates including alpha‐fetoprotein (AFP), macrovascular invasion, extrahepatic spread, ECOG performance status, Child‐Pugh score,^2^, ^11^ MELDNa score,^12^ age, and history of hospitalization for hepatic decompensation in the prior 6 months^13^ were pre‐specified to be included as clinically important confounders in multivariable analyses. Additional covariates including the history of local therapies, time from diagnosis of HCC to first systemic treatment, viral etiology of cirrhosis,^14^ VA medical center complexity score,^15^ BMI, race, Cirrhosis Comorbidity index,^16^ history of VTE in the prior 5 years, history of myocardial infarction (MI) or ischemic cerebrovascular accident (CVA) in the prior 5 years, and calendar time were assessed due to possible role as confounders between the relationship of treatment with sorafenib or nivolumab and OS. Each covariate was assessed in the same way in nivolumab and sorafenib treatment groups. History of autoimmune disease was evaluated as a potential predictor of treatment choice but was thought unlikely to be associated with the outcome of OS in this clinical scenario and was not prespecified as a potential confounder.

Safety outcomes included hospitalization for hepatic decompensation,^13^, ^17^, ^18^ hospitalization for VTE,^19^ hospitalization for GI bleeding,^18^ and hospitalization for CVA or MI.^20^, ^21^ Additional covariates were assessed due to their potential role as confounders of the relationship of treatment choice with safety outcomes and are listed in the Supplementary Material (Table S1). Full definitions and methods of assessment for covariates and outcomes are detailed in the Appendix S1, including ICD codes used to identify secondary outcomes (Table S2). The reason for discontinuation of therapy was assessed by chart review in all patients who received nivolumab and in a propensity score‐matched cohort of patients who received sorafenib with the methods, including statistical analysis, outlined in the Appendix S1.

### 2.3 Statistical analysis

All analyses were performed in Stata (Release 17; College Station, TX). All statistical testing used was two‐sided with an alpha of 0.05. Summary statistics were calculated for baseline covariates in the overall cohort and within sorafenib and nivolumab treatment groups. Univariable survival analysis was performed on the overall cohort and within sorafenib and nivolumab treatment groups using the Kaplan–Meier method with log‐rank test and univariable Cox proportional hazards model. Exploratory analysis examined the unadjusted association of nivolumab treatment with OS within prognostically important subgroups.

Missing observations for covariates were assumed to be missing at random (MAR) and multiple imputations with chained equations with 30 imputations was used to address missing covariates for all analyses that incorporated covariates. Most covariates had limited missing values (<15%) with the exception of ECOG performance status, macrovascular invasion, extrahepatic spread, and the Cirrhosis Comorbidity Index. The primary analysis was a multivariable Cox proportional hazards model examining the association of first systemic treatment with nivolumab (vs. sorafenib) with OS with multivariable adjustment. A set of covariates was prespecified to be included in the multivariable model, as previously outlined, and the remaining candidate covariates were selected using a backward selection criterion of *p* < 0.15 (Multivariable model). Assessment of all Cox proportional hazards model assumptions are outlined in the Appendix S1.

In addition to the primary analysis, a secondary analysis was performed with a Cox proportional hazards model with inverse probability of treatment weighting (IPTW) based on the propensity score. Methodology for the IPTW model and several sensitivity analyses are outlined in the Appendix S1.

Binary safety outcomes were reported as the proportion of patients experiencing the outcome while on first systemic treatment and comparisons between treatment groups were made using Fisher's exact test. Binary outcomes were also assessed using logistic regression modeling to adjust for pre‐specified potential confounding variables (Table S1).

## 3 RESULTS

### 3.1 Patient and treatment characteristics

**FIGURE 1:**
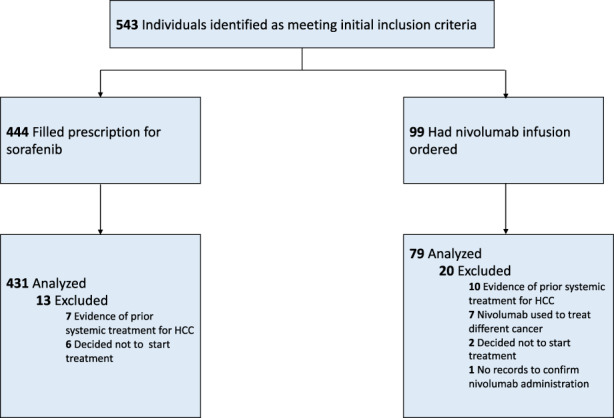
Patient flow diagram

During the study period, 543 patients with HCC and Child‐Pugh B cirrhosis underwent their first systemic treatment. Four hundred and thirty one patients treated with sorafenib and 79 patients treated with nivolumab were included in the analysis (Figure 1). In the overall cohort, 57%/31%/12% had Child‐Pugh B7/B8/B9 cirrhosis, 7% had hepatic encephalopathy, 39% had ascites, 52% had received prior local therapy, 28% had ECOG performance status ≥2, 26% had macrovascular invasion, and 28% had extrahepatic spread (Table 1). The nivolumab cohort had higher proportions of patients with Child‐Pugh B9 cirrhosis, hepatic encephalopathy, ECOG performance status ≥2, macrovascular invasion, extrahepatic spread, CVA or MI in the prior 5 years, VTE in the prior 5 years, variceal bleeding in the prior 5 years, treatment at more complex VA medical centers, and higher median AFP compared to the sorafenib cohort (Table 1). Median duration of treatment in the sorafenib cohort was 48 days (IQR 30–104 days) and 66 days (IQR 21–144 days) in the nivolumab cohort. In the sorafenib cohort, 95 patients (22%) received subsequent systemic therapy (5% subsequently received a tyrosine kinase inhibitor; 20% subsequently received immunotherapy) compared to nine patients (11%) in the nivolumab cohort (10% subsequently received a tyrosine kinase inhibitor; 1% subsequently received immunotherapy).

**TABLE 1:** Baseline patient characteristics

	Total	Sorafenib	Nivolumab	*p*‐value^a^
	*N* = 510	*N* = 431	*N* = 79	
Age—median(IQR)	68.2 (63.9 to 71.8)	68.5 (64.1 to 72.0)	66.6 (63.1 to 71.4)	0.12
Gender—no. (%)				0.81
Female	8 (2%)	7 (2%)	1 (1%)	
Male	502 (98%)	424 (98%)	78 (99%)	
Race—no. (%)				0.53
Black	111 (22%)	93 (22%)	18 (23%)	
Other	87 (17%)	77 (18%)	10 (13%)	
White	312 (61%)	261 (61%)	51 (65%)	
BMI—median(IQR)^b^	27.6 (24.2 to 31.5)	27.6 (24.3 to 31.4)	27.9 (23.8 to 32.1)	0.82
MELDNa—median(IQR)	12.0 (10.0 to 16.0)	12.0 (10.0 to 16.0)	12.0 (9.0 to 15.0)	0.35
Child‐Pugh Score—no. (%)				0.39
7	291 (57%)	247 (57%)	44 (56%)	
8	158 (31%)	136 (32%)	22 (28%)	
9	61 (12%)	48 (11%)	13 (16%)	
Hepatic Encephalopathy—no. (%)				0.02*
Yes	34 (7%)	24 (6%)	10 (13%)	
Ascites—no. (%)				0.78
Yes	201 (39%)	171 (40%)	30 (38%)	
Viral Etiology Cirrhosis—no. (%)	318 (62%)	264 (61%)	54 (68%)	0.23
Etiology of Cirrhosis—no. (%)				0.43
Alcohol‐associated	77 (15%)	64 (15%)	13 (16%)	
Alcohol and Hepatitis C	154 (30%)	126 (29%)	28 (35%)	
Hepatitis B	9 (2%)	7 (2%)	2 (3%)	
Hepatitis C	155 (30%)	131 (30%)	24 (30%)	
NAFLD/NASH	107 (21%)	97 (23%)	10 (13%)	
Other^c^	8 (2%)	6 (1%)	2 (3%)	
AFP—median(IQR)^d^	76.1 (9.7 to 1210.0)	70.1 (9.1 to 1091.0)	171.6 (10.8 to 2980.3)	0.27
Hospitalization for Hepatic decompensation (prior 6 months)—no. (%)				0.83
Yes	108 (21%)	92 (21%)	16 (20%)	
Prior local therapy—no. (%)				0.85
Yes	266 (52%)	224 (52%)	42 (53%)	
Time from diagnosis to first systemic treatment				0.25
≥ Median (7.4 months)	260 (51%)	215 (50%)	45 (57%)	
ECOG performance status—no. (%)				0.61
ECOG 0–1	237 (46%)	195 (45%)	42 (53%)	
ECOG ≥2	141 (28%)	113 (26%)	28 (35%)	
Missing	132 (26%)	123 (29%)	9 (11%)	
Macrovascular invasion—no. (%)				0.063
Yes	131 (26%)	104 (24%)	27 (34%)	
Missing	124 (24%)	106 (25%)	18 (23%)	
Extrahepatic spread—no. (%)				0.26
Yes	142 (28%)	116 (27%)	26 (33%)	
Missing	112 (22%)	95 (22%)	17 (22%)	
VA Complexity Score—no. (%)				0.047*
1a	251 (49%)	204 (47%)	47 (59%)	
1b/1c/2/3	259 (51%)	227 (53%)	32 (41%)	
Calendar Year—no. (row %)				<0.001***
2017	49	47 (96%)	2 (4%)	
2018	198	176 (89%)	22 (11%)	
2019	119	102 (86%)	17 (14%)	
2020	106	77 (73%)	29 (27%)	
2021	38	29 (76%)	9 (24%)	
History of CVA or MI (prior 5 years)—no. (%)				0.015*
Yes	33 (6%)	23 (5%)	10 (13%)	
History of VTE (prior 5 years)—no. (%)				0.24
Yes	110 (22%)	89 (21%)	21 (27%)	
History of Variceal Bleeding (prior 5 years)—no. (%)				0.093
Yes	89 (17%)	70 (16%)	19 (24%)	

Abbreviations: AFP, alpha‐fetoprotein; BMI, body mass index; CVA, cerebrovascular accident (stroke); ECOG, Eastern Cooperative Oncology Group; IQR, inter‐quartile range; MELDNa, model for end stage liver disease plus sodium score; MI, myocardial infarction; NAFLD/NASH, non‐alcohol associated fatty liver disease or steatohepatitis; VTE, venous thromboembolism.

^a^ Categorical and binary variables are compared across treatment groups using Pearson's chi‐square test. Continuous variables are compared across treatment groups using two‐sample, two‐sided t‐tests for normally distributed variables or Wilcoxon rank sum tests for non‐normally distributed variables.

^b^ 7.44% of individuals had missing data for BMI.

^c^ Includes autoimmune hepatitis, primary biliary cirrhosis, and cryptogenic cirrhosis.

^d^ 10.37% of individuals had missing data for AFP.

### 3.2 Univariable survival analysis

**FIGURE 2:**
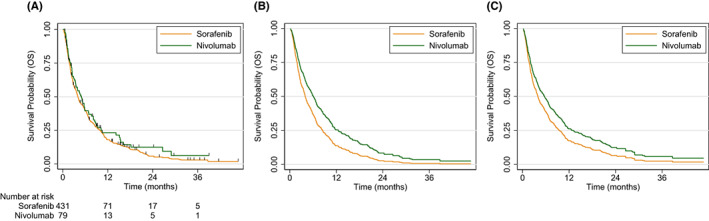
(A) Univariable Kaplan Meier analysis, (B) predicted survival functions based on the final multivariable Cox proportional hazards model for hypothetical patients receiving sorafenib and nivolumab treatments with all covariates set at the mean values, and (C) predicted survival functions based on the Cox proportional hazards model with Inverse Probability of Treatment Weighting (IPTW) for a hypothetical group of patients receiving sorafenib and nivolumab treatments. Predicted survival functions in panels (B) and (C) are for hypothetical groups of patients; thus, the number at risk cannot be calculated.

Death was observed in 457 patients (90%). In the overall cohort, median OS was 4.1 months (95% CI 3.6–4.8). Median OS was 4.0 months (95% CI 3.5–4.8) in the sorafenib cohort and 5.0 months (95% CI 3.3–6.8) in the nivolumab cohort (HR 0.86, 95% CI 0.66–1.13; p = 0.3) (Figure 2). One‐year OS was 18% (95% CI 15–22) in the sorafenib cohort and 23% (95% CI 14–34) in the nivolumab cohort. Exploratory, unadjusted subgroup analysis suggested possible increased benefit to nivolumab treatment in patients with poor performance status (Figure S1).

### 3.3 Multivariable survival analysis

In the primary analysis, treatment with nivolumab was associated with 31% reduced hazard of death compared to treatment with sorafenib (HR 0.69, 95% CI 0.52–0.91; *p* = 0.008) after adjusting for age, race, Child‐Pugh score, log‐transformed AFP, MELDNa, time from diagnosis to first systemic therapy, ECOG performance status, Cirrhosis Comorbidity Index, macrovascular invasion, extrahepatic spread, hospitalization for hepatic decompensation in the prior 6 months, and VA complexity score (Table 2; Table S3) The proportional hazards assumption and linearity assumption for continuous covariates were met in this model. In the secondary analyses, a propensity score model including the same covariates as the multivariable model (with the exceptions of the exclusion of the Cirrhosis Comorbidity Index and the inclusion of viral etiology of cirrhosis, 5‐year history of VTE, and 5‐year history of CVA or MI) led to adequate balance of covariates after IPTW as assessed by standardized differences between −0.1 and 0.1 for prespecified confounding variables (Table S4). The direction and magnitude of the estimated association of nivolumab treatment with OS in the IPTW analysis was similar but did not reach statistical significance (HR 0.77, 95% CI 0.55–1.06; *p* = 0.11) (Table 2). Sensitivity analyses that evaluated the positivity assumption in the IPTW analysis and evaluated the impact of differential missingness in ECOG performance status in the multivariable model did not have a significant impact on the effect estimates (Table S5).

**TABLE 2:** Summary of hazard ratio for overall survival outcome for nivolumab compared to sorafenib across different analyses

	Univariable model	Multivariable model	IPTW model
HR (95% CI)	0.86 (0.66–1.13)	0.69 (0.52–0.91)	0.77 (0.55–1.06)

Abbreviations: CI, confidence interval; HR, hazard ratio; IPTW, inverse probability of treatment weighting.

### 3.4 Safety outcomes

When comparing patients who received nivolumab to patients who received sorafenib in unadjusted analysis, there was no statistically significant difference in the proportion of patients experiencing hospitalization for hepatic decompensation (22% vs. 16%; *p* = 0.25), hospitalization for GI bleeding (9% vs. 8%, *p* = 0.83), or hospitalization for CVA or MI (0% vs. 4%; *p* = 1.0) (Table 3). However, patients who received nivolumab were more likely to have experienced hospitalization for VTE during the first systemic therapy compared to patients who received sorafenib (16% vs. 9%; *p* = 0.04). In multivariable logistic regression modeling, there was no significant difference between patients treated with nivolumab and patients treated with sorafenib in odds of hospitalization for hepatic decompensation (OR 1.03; 95% CI 0.49–2.14; *p* = 0.95), hospitalization for VTE (OR 1.75; 95% CI 0.82–3.75; *p* = 0.15), or hospitalization for GI bleeding (OR 1.03; 95% CI 0.42–2.52; *p* = 0.95) (Table 3). Hospitalization for CVA or MI was not assessed in multivariable modeling due to insufficient number of outcomes observed.

**TABLE 3:** Safety outcomes by treatment group in unadjusted analyses and after multivariable adjustment

	Nivolumab—no. (%)	Sorafenib—no. (%)	Unadjusted analysis ‐ OR for nivolumab compared to sorafenib (95% CI)	Multivariable analysis ‐ OR for nivolumab compared to sorafenib (95% CI)
Hospitalization for hepatic decompensation	17 (22%)	69 (16%)	1.44 (0.79–2.60)	1.03 (0.49–2.14)
Hospitalization for venous thromboembolism	13 (16%)	37 (9%)	2.10 (1.06–4.15)	1.75 (0.82–3.75)
Hospitalization for GI bleeding (including variceal)	7 (9%)	36 (8%)	1.08 (0.46–2.49)	1.03 (0.42–2.52)
Hospitalization for ischemic stroke or myocardial infarction	0 (0%)	4 (1%)	NA^a^	NA^a^

Abbreviations: CI, confidence interval; NA, not applicable; OR, odds ratio.

^a^ Insufficient number of events to estimate OR or perform multivariate modeling.

For the analysis evaluating reason for discontinuation of therapy, propensity score‐matched cohorts (78 patients treated with sorafenib and 78 patients treated with nivolumab) demonstrated adequate balance of baseline patient characteristics with most variables having standardized differences between −0.1 and 0.1, though this was not possible for all variables (Table S6). Patients treated with nivolumab were significantly less likely than patients treated with sorafenib to discontinue treatment due to toxicity (12% vs. 36%; *p* = 0.001) (Table 4). However, when comparing rates of discontinuation for toxicity or clinical decline not directly attributable to therapy, there was no statistically significant difference between patients who received nivolumab and those who received sorafenib (36% vs. 47%; *p* = 0.20). There was no difference between nivolumab and sorafenib cohorts in the proportion of patients treated until disease progression (29% vs. 21%; *p* = 0.27). In the nivolumab group, 5% of patients remained on treatment or discontinued due to long‐term stability compared to zero patients in the sorafenib group (*p* = 0.12). In patients who discontinued treatment due to toxicity, specific reasons are categorized in Table 4.

**TABLE 4:** Categorization of reason for discontinuation of therapy by treatment group

Reason for discontinuation	Nivolumab (*N* = 78)^a^	Sorafenib (*N* = 78)^a^
Death—no. (%)	15 (19%)	18 (23%)
Clinical decline—no. (%)	19 (24%)	9 (12%)
Toxicity—no. (%)	9 (12%)	28 (36%)
Rash—no. (%)	NA^b^	4 (14%)
Bleeding—no. (%)	NA^b^	2 (7%)
GI—no. (%)	NA^b^	9 (32%)
Fatigue—no. (%)	NA^b^	4 (14%)
Autoimmune—no. (%)	6 (67%)	NA^c^
Other—no. (%)	3 (33%)	15 (54%)
Disease progression—no. (%)	23 (29%)	16 (21%)
Patient preference—no. (%)	1 (1%)	1 (1%)
Remains on treatment—no. (%)	3 (4%)	0 (0%)
Prolonged disease stability—no. (%)	1 (1%)	0 (0%)
Lost to follow up—no. (%)	7 (9%)	6 (8%)

Abbreviations: NA, not applicable.

^a^ One patient from nivolumab group remained unmatched and was not included in the reason for discontinuation analysis.

^b^ Rash, bleeding, GI toxicity, and fatigue were not directly assessed as reason for discontinuation for nivolumab patients.

^c^ Autoimmune toxicity not applicable for patients who received sorafenib.

## 4 DISCUSSION

We found that in a retrospective cohort of patients with HCC and Child‐Pugh B cirrhosis treated at VA medical centers, nivolumab treatment was associated with a 31% reduction in hazard of death compared to sorafenib treatment in multivariable analysis. While the effect direction and magnitude were similar in IPTW analysis (23% reduction in hazard of death), the result was not statistically significant. To put the magnitude of the impact on overall survival in context, the HIMALAYA trial recently reported a 22% reduction in hazard of death with tremelimumab plus durvalumab treatment compared to sorafenib in patients with Child‐Pugh A cirrhosis with FDA approval expected on the basis of these results.^22^ In conjunction with safety analyses demonstrating similar rates of hospitalization for key adverse events and lower likelihood of discontinuing treatment due to toxicity, these results support nivolumab as a first‐line treatment option in patients with HCC and Child‐Pugh B cirrhosis.

Secondary analysis with IPTW did not demonstrate a statistically significant association of nivolumab treatment with OS. The reason for the lack of statistical significance in the IPTW analysis may be due to limited power, as our current sample size has only 49% power to detect a true hazard ratio of 0.77 for OS of nivolumab compared to sorafenib, while the same samples size is associated with a power of 81% to detect a true hazard ratio of 0.69 as was observed in the multivariable analysis. However, the direction and magnitude of effect sizes across multiple methods used to adjust for confounding and sensitivity analyses are largely consistent and provide further confidence that the results are robust across statistical methodologies.

Rates of discontinuation of therapy due to adverse events or toxicity in prior observational studies of patients with Child‐Pugh B cirrhosis range from 12%–40% for sorafenib^7^, ^23^, ^24^ and 1.4%–22% for nivolumab.^25^, ^26^ In the present study, 36% of patients in the sorafenib cohort discontinued treatment due to toxicity compared to 12% of nivolumab patients (*p* = 0.001). As some of the primary toxicities of sorafenib (fatigue, anorexia, nausea) overlap with symptoms of disease progression or liver dysfunction and can be misattributed, we examined the combined outcome of discontinuation of therapy for clinical decline or toxicity. There was a trend towards lower rates of discontinuation for toxicity or clinical decline with nivolumab compared to sorafenib, though this was not statistically significant (36% vs. 47%; *p* = 0.20). Additionally, 4% of patients remained on treatment and one patient had treatment discontinued due to long‐term disease stability in the nivolumab cohort compared to zero patients for each of these categories in the sorafenib cohort. While subgroup analyses are purely exploratory in our study given the small sample size in the nivolumab group and lack of adjustment for confounding, Figure S4 generates the hypothesis that patients with poor performance status may particularly benefit from nivolumab rather than sorafenib, perhaps due to better tolerability of nivolumab. The overall profile of safety outcomes in the present study suggests that nivolumab may be more tolerable than sorafenib in patients with Child‐Pugh B cirrhosis.

Recently, the accelerated approval for nivolumab as treatment for patients with HCC was withdrawn by the FDA given lack of confirmatory data in phase III studies.^27^ Durvalumab, a PD‐L1 inhibitor, was recently shown to be statistically non‐inferior to sorafenib in the phase III HIMALAYA trial in patients with HCC and Child‐Pugh A cirrhosis and FDA approval for durvalumab as a first‐line therapy option for HCC is anticipated.^22^ Durvalumab demonstrated similar efficacy to that observed with nivolumab in CheckMate‐459, with effect sizes compared to sorafenib (HR 0.86 and HR 0.85) and overall response rates (17% vs. 15%) being nearly identical. Additionally, durvalumab and nivolumab have similar safety profiles. Given the similar mechanisms of action (preventing PD‐1 and PD‐L1 interaction) and similar safety and efficacy profiles in patients with Child‐Pugh A cirrhosis, it is reasonable to expect similar safety and efficacy of durvalumab as has been observed with nivolumab in patients with Child‐Pugh B cirrhosis. Future prospective trials should evaluate the use of durvalumab in this patient population.

Sorafenib should continue to have an important role in patients with HCC and Child‐Pugh B cirrhosis. It remains an option for use as first systemic therapy, especially for patients with relative or strong contraindications to immune checkpoint inhibitor therapy, such as those with a history of a liver transplant. Additionally, a significant proportion of patients (20.4% in our study) receive subsequent systemic therapy and sorafenib and nivolumab remain the only agents with a well‐established safety profile in patients with Child‐Pugh B cirrhosis. Patients who are eligible for subsequent lines of therapy will have the opportunity to benefit both from sorafenib and immune checkpoint inhibitors, such as nivolumab and durvalumab.

Strengths of the study include that we used a large, national cohort of patients treated in a real‐world setting using a common data source to allow for the first large head‐to‐head comparison of treatment with sorafenib and nivolumab in patients with Child‐Pugh B cirrhosis. This approach fills a critical knowledge gap that exists due to the exclusion of patients with Child‐Pugh B cirrhosis from clinical trials due to their propensity for adverse events and overall poor prognosis. Registrational trials of systemic therapy in patients with HCC have either excluded or accrued only small numbers of patients with Child‐Pugh B cirrhosis.^3^, ^4^, ^5^, ^6^ Prior studies demonstrated safety in patients with HCC and Child‐Pugh B cirrhosis treated with sorafenib or nivolumab, but no prior study has compared OS with robust adjustment for confounding.^7^, ^8^, ^23^, ^24^, ^25^, ^26^, ^28^, ^29^, ^30^ While a randomized trial comparing treatment with nivolumab (or durvalumab) to sorafenib in patients with HCC with Child‐Pugh B cirrhosis would be the gold standard for defining the effect of this treatment choice on OS, high‐quality observational data with robust adjustment for confounding will likely remain the best data available to drive clinical decisions in this setting.

Limitations of the study include that multivariable adjustment or propensity score methods cannot adjust for unmeasured confounding, and we cannot rule out the presence of unmeasured confounding.^31^ However, we were able to adjust for a robust collection of clinically important covariates, including HCC‐specific factors, multiple measures of liver function, patient‐specific factors, and provider and medical center‐specific factors. A second limitation is that the sample size, particularly within the nivolumab cohort, remains small and limits the number of covariates that can be adjusted for within a multivariable model or within a propensity score model without biasing the observed effect sizes.^32^ Third, reliable data on the cause of death in our cohort was not available. This would have provided information to help investigate the mechanism for reduced hazard of death in the nivolumab cohort, such as reduced toxicity and death related to decompensated cirrhosis versus reduced death due to improved tumor control. Finally, the generalizability of the study is limited due to the cohort consisting of patients treated at VA medical centers, with very few female patients or patients with hepatitis B.

In patients with HCC and Child‐Pugh B cirrhosis treated in a real‐world setting, nivolumab treatment was associated with improved overall survival after adjusting for clinically important covariates in multivariable analysis and less discontinuation of treatment due to toxicity compared to sorafenib. PD‐1/PD‐L1 inhibitors such as nivolumab should be considered as a first‐line treatment option in this patient population.

## AUTHOR CONTRIBUTIONS

William J. Chapin: Conceptualization, methodology, data curation, formal analysis, visualization, writing—original draft, and writing—review and editing. Wei‐Ting Hwang: Conceptualization, methodology, formal analysis, and writing—review and editing. Thomas B. Karasic: Conceptualization, methodology, supervision, and writing—review and editing. Anne Marie McCarthy: Conceptualization, methodology, formal analysis, visualization, supervision, and writing—review and editing. David E. Kaplan: Conceptualization, methodology, data curation, formal analysis, visualization, resources, software, supervision, and writing—review and editing.

## FUNDING INFORMATION

This work was supported by the National Institutes of Health (T32 CA009679 to WJC).

## CONFLICT OF INTEREST

David E. Kaplan—Grant support to Institution: Gilead Sciences, Bayer Healthcare Inc, and AstraZeneca Inc. Thomas B. Karasic—Honoraria/Advisory Board: Pfizer, Exelixis, Incyte, Ipsen, AstraZeneca, Genentech; Research Support: BMS (unrelated to HCC), Eli Lilly, Xencor, Taiho, Tempest, H3Biomedicine. No other disclosures were reported.

## ETHICS STATEMENT

Ethics approval was sought and the study was deemed exempt by the Institutional Review Board at the Corporal Michael J. Crescenz VA Medical Center.

## Supporting information


Appendix S1

## Data Availability

The data underlying this article will be released upon reasonable request in a de‐identified fashion after a Data Use Authorization or Data Transfer Agreement is made with the Corporal Michael J. Crescenz VA Medical Center.
