# Supplementary material for: Comparison of nivolumab and sorafenib for first systemic therapy in patients with hepatocellular carcinoma and Child‐Pugh B cirrhosis
Source: Cancer Med. 2022 Jun 2;12(1):189–99. doi: 10.1002/cam4.4906 (PMC9844625; doi:10.1002/cam4.4906)
Supplement: Supplementary file 1 — Appendix S1 [file CAM4-12-189-s001.docx]

**Supplementary Material**

[Supplementary Methods 2](#_Toc92890094)

[Definitions of covariates 2](#_Toc92890095)

[Definitions of outcomes 3](#_Toc92890096)

[Categorization of reason for discontinuation of therapy 4](#_Toc92890097)

[Assessment of assumptions for all Cox proportional hazards models 5](#_Toc92890098)

[Statistical methods for propensity score analyses for overall survival outcome 5](#_Toc92890099)

[Sensitivity analyses 5](#_Toc92890100)

[Statistical methods for reason for discontinuation outcome 5](#_Toc92890101)

[Supplementary Table 1: Pre-specified multivariable models for safety outcomes. 6](#_Toc92890102)

[Supplementary Table 2: ICD-9 and ICD-10 codes for covariates and outcomes of interest. 6](#_Toc92890103)

[Supplementary Table 3: Multivariable Cox proportional hazards model for overall survival outcome. 8](#_Toc92890104)

[Supplementary Table 4: Balance of clinically important covariates in unadjusted analysis and after inverse probability of treatment weighting (IPTW). 9](#_Toc92890105)

[Supplementary Table 5: Effect estimates of treatment choice on overall survival after sensitivity analyses 10](#_Toc92890106)

[Supplementary Table 6: Balance of baseline covariates across treatment groups after propensity score matching for the reason for discontinuation of therapy analysis. 10](#_Toc92890107)

[Supplementary Figure 1: Unadjusted, exploratory analysis examining hazard ratio for nivolumab compared to sorafenib for overall survival within prognostically important subgroups. 12](#_Toc92890108)

[Supplementary References: 12](#_Toc92890109)

Supplementary Methods

Definitions of covariates

Alpha-fetoprotein (AFP): Lab result closest to, but prior to, first systemic treatment date. Treated with a log transformation due to right skew and to ensure linearity assumption met in Cox proportional hazards modeling.

History of local therapy: Any procedure code for hepatectomy, ablation, radiation, or embolization prior to date of first systemic treatment. Coded as a binary variable as present or absent.

Time from diagnosis to first systemic treatment: Difference in days between first diagnostic code for hepatocellular carcinoma and the date of first systemic treatment. Was classified as a binary variable with a cut point at the median (224 days).

Macrovascular invasion: Gross tumor invasion of the portal vein or hepatic vein branches mentioned in the report for MRI abdomen, CT abdomen, or PET CT within 60 days prior to systemic treatment initiation. Criteria were only met if the report specifically mentioned features specific for tumor thrombus, tumor in vein, or vascular invasion, or that findings were suspicious for tumor thrombus, tumor in vein, or vascular invasion. If portal vein or hepatic vein thrombosis was mentioned without commentary on likelihood of representing tumor thrombus or tumor in vein, the observation was coded as 0, or no macrovascular invasion. Coded as a binary variable with categories for present and absent. If not evaluable, then was coded as missing.

Extrahepatic spread: Evidence of sites of disease outside of the liver (including perihepatic lymph nodes) on imaging reports that occurred within 60 days prior to systemic treatment initiation. Specifically, sites were recorded as representing a site of extrahepatic disease if >1 cm in diameter for tumor metastases or implants and the imaging report mentioned that the site possibly, or likely, represented metastatic disease. To be considered a site of extrahepatic disease, lymph nodes needed to be at least 1.5cm in short axis or 3cm in long axis with commentary that they possibly, or likely, represented sites of cancer involvement. Patients were considered assessable for this covariate (not missing) if they had at least abdominal imaging within the time frame above, even if no chest imaging was performed during the appropriate time window. Sites of extrahepatic disease did not need to meet size criteria noted above if they were biopsy proven prior to the date of first systemic treatment. Coded as a binary variable with categories for present or absent. If not evaluable, then was coded as missing.

Child-Pugh Score: Calculated as previously validated in VA database with the modification that points were subtracted for INR elevation if patient was concurrently on anticoagulation therapy.^1^ For example, if patient received three points for INR elevation to 2.5 while on anticoagulation and had a Child-Pugh score of C10, two points would be subtracted and the final calculated Child-Pugh score would be B8. Treated as a three-level categorical variable for Child-Pugh 7, 8, or 9.

ECOG performance status: Determined from clinical notes within 30 days prior to first systemic treatment date. Was coded as specifically recorded in the clinic note if available. If ECOG performance status was not specifically recorded but could be inferred from information available within the clinical notes, it was inferred. If Karnofsky performance status or palliative performance scale were recorded, these were translated to an ECOG performance status.^2–4^ This was subsequently categorized as a binary variable as ECOG < 2 and ECOG ≥ 2. If not evaluable, was coded as missing.

MELDNa score: Calculated using lab data collected prior to first systemic treatment date.^5^ Treated as an ordinal variable.

History of hospitalization for hepatic decompensation in prior 6 months: One inpatient diagnostic code for ascites, spontaneous bacterial peritonitis, esophageal variceal bleeding, hepatic encephalopathy, and hepatorenal syndrome as outlined in Supplementary Table 1.^6–8^

Viral etiology of cirrhosis: Etiology of cirrhosis was defined per previously validated algorithm.^9^ Etiology was subsequently categorized as viral etiology of cirrhosis if defined as an HCV or HBV cirrhosis category and as non-viral cirrhosis if grouped in any other cirrhosis categories. This categorization was chosen based on recent work examining the efficacy of immunotherapy in viral and non-viral etiologies of cirrhosis.^10^

VA medical center complexity score: Published by the Veterans Administration to categorize the overall clinical complexity of VA medical centers including patient population treated, clinical services complexity, and education and research missions of facilities. Includes five levels including 1a (most complex), 1b, 1c, 2, and 3 (least complex).^11^ We categorized as a binary variable as most complex (1a) and less complex (1b/1c/2/3) to achieve similar numbers of patients within each level of the binary variable.

Age: Reported in years and treated as a continuous variable. Age divided by 5 was used to report effect sizes for 5-year increments in age to aid in interpretability.

Body mass index (BMI): Treated as a continuous variable.

Race: As recorded within the Corporate Data Warehouse as White, Black, Asian, Pacific Islander, Native American, Unknown, and Other. We categorized as White, Black, or Other by grouping the remaining categories to best balance the number of patients in each category.

Gender: As recorded within the VA Corporate Data Warehouse.

Cirrhosis Comorbidity Index (CirCom index): Calculated per previously validated method and treated as an ordinal variable.^12^

History of venous thromboembolism in the prior 5 years: Defined by one inpatient or two outpatient diagnostic codes (Supplementary Table 1) for deep venous thrombosis, pulmonary embolism, or portal vein thrombosis in the 5 years prior to first systemic treatment.^13^

History of myocardial infarction (MI) or ischemic cerebrovascular accident (CVA) in the prior 5 years: Defined by one inpatient or two outpatient diagnostic codes for acute MI or ischemic CVA. ^14,15^

Calendar time: Treated as a binary variable with cutoff determined by median date of first systemic treatment (01/29/2019).

Anticoagulation use: Outpatient use of systemic anticoagulation in the 30-day window prior to first systemic treatment date. Coded as a binary variable.

Antiplatelet use: Outpatient use of antiplatelet medication in the 30-day window prior to first systemic treatment date. Coded as a binary variable.

Hepatic encephalopathy medication use: Outpatient use of hepatic encephalopathy medications including lactulose and/or rifaximin in the 30-day window prior to first systemic treatment date. Coded as a binary variable.

Ascites medications use: Outpatient use of diuretic medications used for ascites such as furosemide and spironolactone in the 30-day window prior to first systemic treatment date. Coded as a binary variable.

Definitions of outcomes

Overall survival: Time from date of first systemic treatment to documented date of death, or censoring at last VA follow up or administrative censoring on November 15, 2021, whichever occurred first.

Hospitalization for hepatic decompensation: One inpatient diagnostic code for ascites, spontaneous bacterial peritonitis, esophageal variceal bleeding, hepatic encephalopathy, and hepatorenal syndrome as outlined in Supplementary Table 1.^6–8^ Patient was considered at-risk for the outcome only during first systemic treatment. Coded as a binary variable (0 if event did not occur; 1 if event occurred during first systemic treatment) rather than a count variable or time-to-event variable as repeat events were rare and duration of first systemic treatment was similar between treatment groups.

Hospitalization for venous thromboembolism: Defined by one inpatient diagnostic code (Supplementary Table 1) for deep venous thrombosis, pulmonary embolism, or portal vein thrombosis.^13^ Patient was considered at-risk for the outcome only during first systemic treatment. Coded as a binary variable (0 if event did not occur; 1 if event occurred during first systemic treatment) rather than a count variable or time-to-event variable as repeat events were rare and duration of first systemic treatment was similar between treatment groups.

Hospitalization for GI bleeding: Defined by one inpatient diagnostic code for variceal bleeding or other GI bleeding (Supplementary Table 1).^7^ Patient was considered at-risk for the outcome only during first systemic treatment. Coded as a binary variable (0 if event did not occur; 1 if event occurred during first systemic treatment) rather than a count variable or time-to-event variable as repeat events were rare and duration of first systemic treatment was similar between treatment groups.

Hospitalization for ischemic CVA or MI: Defined by one inpatient diagnostic code for MI or ischemic CVA (Supplementary Table 1).^14,15^ Patient was considered at-risk for the outcome only during first systemic treatment. Patient was not considered at risk after experiencing death or discontinuation of therapy. Coded as a binary variable (0 if event did not occur; 1 if event occurred during first systemic treatment) rather than a count variable or time-to-event variable as repeat events were rare and duration of first systemic treatment was similar between treatment groups.

Categorization of reason for discontinuation of therapy

Reason for discontinuation of therapy: Coded as a categorical variable (based on reason for discontinuation reported in clinician notes) with categories including death, disease progression, toxicity, clinical decline, patient preference, loss to follow up, patient remained on therapy, or discontinued due to ongoing disease stability. If multiple reasons were given for discontinuation, but included disease progression, then disease progression took precedence.

End of therapy was calculated as 21 days after last nivolumab infusion for nivolumab patients or the end of the last sorafenib prescription (e.g., 30 days after a 30-day prescription for sorafenib was filled). Definition of subcategories of reason for discontinuation follow:

Death: If death occurred prior to calculated end of therapy date.

Toxicity: Clinical documentation of treatment toxicity leading to discontinuation of therapy. This included if patient self-discontinued treatment for a specific mention of toxicity without discussing with clinician. It also included lab abnormalities that were cited as the proximal event leading to discontinuation of therapy. For patients on nivolumab, toxicity was categorized as autoimmune or other. To be classified as autoimmune toxicity, the clinician had to have a high enough suspicion to prescribe systemic steroid therapy. For patients on sorafenib, toxicity was sub-classified as rash, GI toxicity including nausea and diarrhea, fatigue, bleeding, or other.

Clinical decline: This included clinical documentation of decline in clinical status that is common in the Child-Pugh B patient population that was the proximal event leading to discontinuation of anti-cancer therapy. This included inpatient hospitalization where the reason for hospitalization was not directly attributed to anti-cancer therapy.

Disease progression: Clinical documentation of disease progression with imaging progression and/or AFP rise cited as evidence of disease progression. If disease progression occurred concurrently with another reason for discontinuation of therapy, then disease progression was classified as the reason for discontinuation.

Patient preference: Clinical documentation of patient indicating preference for discontinuing treatment for reasons other than treatment intolerance or toxicity. Examples included logistical/travel reasons and philosophy regarding end-of-life care.

Patient remains on treatment: Coded if patient remained on first systemic therapy at the time of data cutoff.

Disease stability: Clinical documentation of discontinuation of systemic therapy due to prolonged disease stability with no evidence of progression at time of treatment discontinuation.

Lost to follow up: No clinical documentation of patient’s therapeutic status for 30-days after the end of treatment date or documentation of patient no-shows to all clinical appointments during this 30-day time window.

Assessment of assumptions for all Cox proportional hazards models

For Cox proportional hazards models, the proportional hazards assumption was assessed using a Schoenfeld global test in individual imputed data sets. Linearity in the log hazard of continuous covariates was assessed by plotting Martingale residuals against each continuous covariate in individual imputed data sets.

Statistical methods for propensity score analyses for overall survival outcome

A propensity score logistic regression model was generated using all candidate covariates proposed to be confounders between the relationship of sorafenib and nivolumab treatment and overall survival. Fit of the logistic regression model was assessed using the Hosmer-Lemeshow goodness of fit test within individual imputed data sets. Stabilized inverse probability weights were generated and covariate balance was assessed after inverse probability of treatment weighting (IPTW) over the 30 imputed data sets using *pbalchk* in Stata.^16,17^ The distribution of continuous covariates by treatment group after IPTW were assessed to ensure appropriate covariate balance for continuous variables. Distribution of the propensity score by treatment group was evaluated to ensure no violation of the positivity assumption. Changes to the propensity score model were permitted to ensure appropriate model fit and appropriate balance of covariates across treatment groups as indicated by standardized differences between -0.1 and 0.1, prioritizing minimization of standardized differences for the pre-specified covariates outline in the *Exposure, outcomes, and covariates* section of the methods. Standardized differences for each covariate across treatment groups were calculated as the difference in means (for continuous variables) or proportions (for binary variables) normalized to the pooled standard deviations.^18^

Once a propensity score model was selected and determined to have appropriate model fit and balance of covariates across treatment groups following IPTW, a Cox proportional hazards model examining overall survival by sorafenib or nivolumab treatment was performed with IPTW (IPTW model).

Sensitivity analyses

For the overall survival outcome examined with the IPTW model, sensitivity analysis was performed which examined the distribution of propensity scores by treatment choice and limited the analysis to areas of overlap between the propensity score distributions in order to ensure that the positivity assumption was met.

A second sensitivity analysis was performed to evaluate the potential impact of imbalanced missingness in ECOG performance status on the effect size measurement. This involved examining an “extreme case” where all of the missing values for ECOG performance status in the sorafenib group were assigned ECOG performance status ≥ 2 while all of the missing values in the nivolumab group were assigned ECOG performance status < 2 and examining the effect size observed in the multivariable model.

Statistical methods for reason for discontinuation outcome

In order to balance baseline treatment characteristics between nivolumab and sorafenib treatment groups for the “reason for discontinuation of therapy” analysis, propensity score matching was performed. This also served to select a sample of patients from the sorafenib group to limit the number of patients that would need data abstraction for the “reason for discontinuation of treatment” outcome. A propensity score was generated using covariates proposed to be associated with nivolumab vs sorafenib treatment and the reason for discontinuation of therapy. Subsequently, 1:1 nearest neighbor matching within a caliper width of 0.2 * standard deviation of the logit of the propensity score was performed with the *psmatch2* command in Stata.^19^ Balance of baseline covariates was assessed using *pbalchk* in Stata.^17^

Reason for discontinuation of therapy was assessed as a categorical variable as discussed above, but also as the proportion of patients discontinuing therapy due to toxicity, the proportion of patients discontinuing therapy due to toxicity or clinical decline, the proportion of patients discontinuing therapy for disease progression, and the proportion of patients still on therapy or discontinuing due to continued clinical response. Comparison of binary outcomes between treatment groups was performed using Fisher’s exact test.

Supplementary Table 1: Pre-specified multivariable models for safety outcomes.

| **Outcome** | **Covariates** |
| --- | --- |
| Hospitalization for hepatic decompensation | Hospitalization for hepatic decompensation in prior 6 months, Child-Pugh score, anticoagulant use, hepatic encephalopathy medication use, ascites medication use, esophageal variceal bleeding in prior 5 years, macrovascular invasion |
| Hospitalization for GI bleeding | Hospitalization for GI bleeding in prior 6 months, anticoagulant use, antiplatelet use, Child-Pugh score, esophageal variceal bleeding in prior 5 years |
| Hospitalization for VTE | Hospitalization for VTE in prior 6 months, anticoagulant use, antiplatelet use, Child-Pugh score, VTE in the prior 5 years |
| Hospitalization for CVA or MI | Not assessed due to rare outcome |

Abbreviations: GI, gastrointestinal; VTE, venous thromboembolism; CVA, cerebrovascular accident (stroke); MI, myocardial infarction.

Supplementary Table 2: ICD-9 and ICD-10 codes for covariates and outcomes of interest.

| **Variable** | **ICD-9** | **ICD-10** |
| --- | --- | --- |
| Ascites | 572.3; 789.5; 789.59 | K76.6; R18.0; R18.8 |
| Spontaneous bacterial peritonitis | 567.0; 567.2; 567.23; 567.8; 567.89; 567.9 | K65.2 |
| Variceal bleeding | 456.0; 456.20 | I85.01; I85.11 |
| Hepatic encephalopathy | 572.2 | G93.40; G93.41; G93.49; K72.91 |
| Hepatorenal syndrome | 572.4 | K76.7 |
| Deep venous thrombosis | 453.0; 453.2; 453.40; 453.41; 453.42; 453.82; 453.84; 453.85; 453.86; 453.87; 453.9 | I82.A19; I82.B19; I82.C19; I82.0; I82.220; I82.290; I82.401; I82.403; I82.409; I82.411; I82.412; I82.413; I82.419; I82.421; I82.422; I82.423; I82.429; I82.431; I82.432; I82.433; I82.439; I82.441; I82.442; I82.443; I82.449; I82.491; I82.492; I82.493; I82.499; I82.4Y1; I82.4Y2; I82.4Y3; I82.4Y9; I82.4Z1; I82.4Z2; I82.4Z3; I82.4Z9; I82.621; I82.622; I82.623; I82.629 |
| Pulmonary embolus | 415.1; 415.11; 415.13; 415.19 | I26.01; I26.02; I26.09; I26.90; I26.92; I26.99 |
| Portal vein thrombosis | 452 | I81. |
| Acute myocardial infarction | 410.0; 410.00; 410.01; 410.02; 410.1; 410.10; 410.11; 410.12; 410.2; 410.20; 410.21; 410.22; 410.3; 410.30; 410.31; 410.32; 410.4; 410.40; 410.41; 410.42; 410.5; 410.50; 410.51; 410.52; 410.6; 410.60; 410.61; 410.62; 410.7; 410.70; 410.71; 410.72; 410.8; 410.80; 410.81; 410.82; 410.9; 410.90; 410.91; 410.92; 411.0 | I21.01; I21.02; I21.09; I21.11; I21.19; I21.21; I21.29; I21.3; I21.4; I22.0; I22.1; I22.2; I22.8; I22.9; I23.0; I23.1; I23.2; I23.3; I23.4; I23.5; I23.6; I23.7; I23.8; I24.0; I24.1; I24.8; I24.9 |
| Ischemic cerebrovascular accident | 434.01; 434.1; 434.10; 434.11; 434.90; 434.91; 436. | I63.00; I63.011; I63.012; I63.013; I63.019; I63.02; I63.031; I63.032; I63.033; I63.039; I63.09; I63.10; I63.111; I63.112; I63.113; I63.119; I63.12; I63.131; I63.132; I63.133; I63.139; I63.19; I63.20; I63.211; I63.212; I63.213; I63.219; I63.22; I63.231; I63.232; I63.233; I63.239; I63.29; I63.30; I63.311; I63.312; I63.313; I63.319; I63.321; I63.322; I63.323; I63.329; I63.331; I63.332; I63.333; I63.339; I63.341; I63.342; I63.343; I63.349; I63.39; I63.40; I63.411; I63.412; I63.413; I63.419; I63.421; I63.422; I63.423; I63.429; I63.431; I63.432; I63.433; I63.439; I63.441; I63.442; I63.443; I63.449; I63.49; I63.50; I63.511; I63.512; I63.513; I63.519; I63.521; I63.522; I63.523; I63.529; I63.531; I63.532; I63.533; I63.539; I63.541; I63.542; I63.543; I63.549; I63.59; I63.6; I63.8; I63.81; I63.89; I63.9 |
| GI bleed | 530.82; 531.00; 531.01; 531.20; 531.21; 531.40; 531.41; 531.60; 531.61; 532.00; 532.01; 532.20; 532.21; 532.40; 532.41; 532.60; 532.61; 533.0; 533.01; 533.20; 533.21; 533.40; 533.41; 533.60; 533.61; 534.00; 534.20; 534.21; 534.40; 534.41; 535.01; 535.11; 535.21; 535.31; 535.41; 535.51; 535.61; 537.83; 578.0; 578.1; 578.9 | K25.0; K25.2; K25.4; K25.6; K26.0; K26.2; K26.4; K26.6; K28.0; K28.2; K28.4; K28.6; K29.01; K29.21; K29.41; K29.51; K29.61; K29.71; K29.81; K29.91; K31.811; K92.0; K92.1; K92.2 |

Abbreviations: ICD, International Statistical Classification of Diseases and Related Health Problems; GI, gastrointestinal.

Supplementary Table 3: Multivariable Cox proportional hazards model for overall survival outcome.

| **Variable** | **Hazard Ratio** | **p-value** | **95% Confidence Interval** |
| --- | --- | --- | --- |
| Nivolumab | 0.69 | 0.008 | 0.52 – 0.91 |
| Age/5 | 1.06 | 0.18 | 0.97 – 1.15 |
| Race |  |  |  |
| Black | 0.81 | 0.10 | 0.63 – 1.04 |
| Other | 0.80 | 0.10 | 0.61 – 1.05 |
| Child-Pugh Score |  |  |  |
| 8 | 1.07 | 0.56 | 0.86 – 1.32 |
| 9 | 1.55 | 0.006 | 1.14 – 2.12 |
| log(AFP) | 1.08 | <0.001 | 1.04 – 1.12 |
| MELDNa | 1.04 | <0.001 | 1.02 - 1.05 |
| Time from diagnosis to first systemic treatment (binary, cutpoint at median) | 0.74 | 0.004 | 0.61 – 0.91 |
| ECOG >= 2 | 1.24 | 0.06 | 1.00 – 1.53 |
| CirCom Index | 1.08 | 0.03 | 1.01 – 1.15 |
| Macrovascular Invasion | 1.09 | 0.48 | 0.86 – 1.40 |
| Extrahepatic Spread | 1.47 | 0.002 | 1.15 – 1.89 |
| Hospitalization for Hepatic Decompensation in prior 6 months | 1.28 | 0.04 | 1.01 – 1.63 |
| Lower VA complexity | 0.73 | 0.002 | 0.60 – 0.89 |

Abbreviations: log(AFP), log transformation of alpha-fetoprotein; MELDNa, Model for End Stage Liver Disease plus Sodium; ECOG, Eastern Cooperative Oncology Group; CirCom Index, Cirrhosis Comorbidity Index; VA, veterans affairs.

Supplementary Table 4: Balance of clinically important covariates in unadjusted analysis and after inverse probability of treatment weighting (IPTW).

| **Variable** | **IPTW Standardized Difference^a^** | **Unweighted Standardized Difference** |
| --- | --- | --- |
| History of ischemic CVA or MI (prior 5 years) | 0.01 | 0.26 |
| History of VTE (prior 5 years) | 0.08 | 0.14 |
| AFP | -0.08 | 0.01 |
| log(AFP) | -0.07 | 0.14 |
| Age | -0.04 | -0.20 |
| BMI^b^ | 0.24 | 0.04 |
| Calendar Time^c^ | 0.39 | 0.37 |
| Child-Pugh Score | 0.02 | 0.10 |
| CirCom Index | 0.01 | 0.09 |
| ECOG ≥ 2 | 0.04 | 0.07 |
| Extrahepatic Spread | -0.06 | 0.15 |
| Hospitalization for Hepatic Decompensation (prior 6 months) | 0.00 | -0.03 |
| History of Local Therapy | -0.04 | 0.02 |
| Lower VA Complexity | 0.03 | -0.24 |
| Macrovascular Invasion | 0.00 | 0.24 |
| MELDNa | -0.05 | -0.13 |
| Race_Black | -0.04 | 0.03 |
| Race_Other | -0.14 | -0.14 |
| Race_White | 0.14 | 0.08 |
| Viral Etiology of Cirrhosis | 0.08 | 0.15 |
| Time from Diagnosis to First Systemic Treatment (binary, cutpoint at median) | 0.03 | 0.14 |

Abbreviations: IPTW, inverse probability of treatment weighting; CVA, cerebrovascular accident (stroke); MI, myocardial infarction; VTE, venous thromboembolism; AFP, alpha-fetoprotein; log(AFP), log transformation of alpha-fetoprotein; BMI, body mass index; CirCom Index, Cirrhosis Comorbidity Index; ECOG, Eastern Cooperative Oncology Group; VA, Veterans Affairs; MELDNa, Model for End Stage Liver Disease Plus Sodium.

^a^A standardized difference was calculated as the difference in means or proportions between treatment groups normalized to the pooled standard deviation. A standardized difference of between -0.1 and 0.1 suggests low risk of residual confounding on that variable.^28^

^b^BMI and calendar time (cut point at median) were excluded from the propensity score model as their inclusion induced imbalance on other, more clinically relevant covariates.

Supplementary Table 5: Effect estimates of treatment choice on overall survival after sensitivity analyses

|  | **Multivariable Model** | **Multivariable Model with “Extreme Case” for ECOG missingness** | **IPTW Model** | **IPTW Model “Positivity Assumption” Analysis** |
| --- | --- | --- | --- | --- |
| HR (95% CI) | 0.69 (0.52 – 0.91) | 0.72 (0.54 – 0.95) | 0.77 (0.55 – 1.06) | 0.84 (0.62 – 1.13) |

Abbreviations: HR, hazard ratio; CI, confidence interval; ECOG, Eastern Cooperative Oncology Group; IPTW, inverse probability of treatment weighting.

Supplementary Table 6: Balance of baseline covariates across treatment groups after propensity score matching for the reason for discontinuation of therapy analysis.

| **Variable** | **Standardized Difference in Propensity Score Matched Cohorts** | **Standardized Difference Prior to Propensity Score Matching** |
| --- | --- | --- |
| 5-year history of autoimmune disease | 0.00 | -0.02 |
| 5-year history of CVA or MI | 0.05 | 0.27 |
| 5-year history of VTE | -0.12 | 0.14 |
| 5-year history of variceal bleeding | -0.03 | 0.19 |
| Age | 0.07 | -0.20 |
| ChildPugh-Score | -0.09 | 0.09 |
| CirCom Index | 0.07 | 0.09 |
| ECOG ≥ 2 | -0.04 | 0.08 |
| Extrahepatic Spread | 0.08 | 0.15 |
| Hospitalization for Hepatic Decompensation in prior 6 months | 0.00 | -0.03 |
| History of Local Therapy | -0.36 | 0.02 |
| Lower VA Complexity Score | -0.13 | -0.25 |
| Macrovascular Invasion | 0.10 | 0.26 |
| MELDNa | -0.01 | -0.13 |
| Race_Black | 0.18 | 0.03 |
| Race_Other | -0.14 | -0.15 |
| Race_White | -0.05 | 0.08 |
| Time from Diagnosis to First Systemic Treatment (binary, cutpoint at median) | -0.28 | 0.14 |
| Viral Etiology of Cirrhosis | 0.00 | 0.15 |
| logAFP | 0.14 | 0.15 |

Abbreviations: CVA, cerebrovascular accident (stroke); MI, myocardial infarction; VTE, venous thromboembolism; CirCom Index, Cirrhosis Comorbidity Index; ECOG, Eastern Cooperative Oncology Group; VA, Veterans Affairs; MELDNa, Model for End Stage Liver Disease plus Sodium; logAFP, log transformation of alpha-fetoprotein.

Supplementary Figure 1: Unadjusted, exploratory analysis examining hazard ratio for nivolumab compared to sorafenib for overall survival within prognostically important subgroups. Abbreviations: AFP, alpha-fetoprotein; ECOG, Eastern Cooperative Oncology Group; No. pts, number of patients; Haz. Ratio, hazard ratio; CI, confidence interval.

Supplementary References:

1. Kaplan DE, Dai F, Aytaman A, et al. Development and Performance of an Algorithm to Estimate the Child-Turcotte-Pugh Score From a National Electronic Healthcare Database. *Clin Gastroenterol Hepatol Off Clin Pract J Am Gastroenterol Assoc*. 2015;13(13):2333-2341.e1-6. doi:10.1016/j.cgh.2015.07.010

2. Karnofsky DA, Abelmann WH, Craver LF, Burchenal JH. The use of the nitrogen mustards in the palliative treatment of carcinoma. With particular reference to bronchogenic carcinoma. *Cancer*. 1948;1(4):634-656. doi:10.1002/1097-0142(194811)1:4<634::AID-CNCR2820010410>3.0.CO;2-L

3. Anderson F, Downing GM, Hill J, Casorso L, Lerch N. Palliative performance scale (PPS): a new tool. *J Palliat Care*. 1996;12(1):5-11.

4. ECOG Performance Status. ECOG-ACRIN. Accessed July 27, 2021. https://ecog-acrin.org/resources/ecog-performance-status

5. Kim WR, Biggins SW, Kremers WK, et al. Hyponatremia and mortality among patients on the liver-transplant waiting list. *N Engl J Med*. 2008;359(10):1018-1026. doi:10.1056/NEJMoa0801209

6. Lo Re V, Lim JK, Goetz MB, et al. Validity of diagnostic codes and liver-related laboratory abnormalities to identify hepatic decompensation events in the Veterans Aging Cohort Study. *Pharmacoepidemiol Drug Saf*. 2011;20(7):689-699. doi:10.1002/pds.2148

7. Kanwal F, Kramer JR, Buchanan P, et al. The Quality of Care Provided to Patients With Cirrhosis and Ascites in the Department of Veterans Affairs. *Gastroenterology*. 2012;143(1):70-77. doi:10.1053/j.gastro.2012.03.038

8. Moon AM, Dominitz JA, Ioannou GN, Lowy E, Beste LA. Use of Antibiotics Among Patients With Cirrhosis and Upper Gastrointestinal Bleeding Is Associated With Reduced Mortality. *Clin Gastroenterol Hepatol*. 2016;14(11):1629-1637.e1. doi:10.1016/j.cgh.2016.05.040

9. Beste LA, Leipertz SL, Green PK, Dominitz JA, Ross D, Ioannou GN. Trends in Burden of Cirrhosis and Hepatocellular Carcinoma by Underlying Liver Disease in US Veterans, 2001–2013. *Gastroenterology*. 2015;149(6):1471-1482.e5. doi:10.1053/j.gastro.2015.07.056

10. Pfister D, Núñez NG, Pinyol R, et al. NASH limits anti-tumour surveillance in immunotherapy-treated HCC. *Nature*. 2021;592(7854):450-456. doi:10.1038/s41586-021-03362-0

11. National Academies of Sciences E, Education D of B and SS and, Integration B on HS, Sciences D on E and P, Environment B on I and the C, Administration C on FSR for VH. *Nature of Veterans Health Administration Facilities Management (Engineering) Tasks and Staffing*. National Academies Press (US); 2019. Accessed October 6, 2021. https://www.ncbi.nlm.nih.gov/books/NBK555777/

12. Jepsen P, Vilstrup H, Lash TL. Development and validation of a comorbidity scoring system for patients with cirrhosis. *Gastroenterology*. 2014;146(1):147-156; quiz e15-16. doi:10.1053/j.gastro.2013.09.019

13. Sanfilippo KM, Wang TF, Gage BF, Liu W, Carson KR. Improving accuracy of International Classification of Diseases codes for venous thromboembolism in administrative data. *Thromb Res*. 2015;135(4):616-620. doi:10.1016/j.thromres.2015.01.012

14. Floyd JS, Blondon M, Moore KP, Boyko EJ, Smith NL. Validation of methods for assessing cardiovascular disease using electronic health data in a cohort of Veterans with diabetes. *Pharmacoepidemiol Drug Saf*. 2016;25(4):467-471. doi:https://doi.org/10.1002/pds.3921

15. Goldstein LB. Accuracy of ICD-9-CM Coding for the Identification of Patients With Acute Ischemic Stroke. *Stroke*. 1998;29(8):1602-1604. doi:10.1161/01.STR.29.8.1602

16. Cole SR, Hernán MA. Constructing Inverse Probability Weights for Marginal Structural Models. *Am J Epidemiol*. 2008;168(6):656-664. doi:10.1093/aje/kwn164

17. Lunt M. Propensity Analysis in Stata Revision: 1. :30.

18. Austin PC, Stuart EA. Moving towards best practice when using inverse probability of treatment weighting (IPTW) using the propensity score to estimate causal treatment effects in observational studies. *Stat Med*. 2015;34(28):3661-3679. doi:https://doi.org/10.1002/sim.6607

19. Leuven E, Sianesi B. PSMATCH2: Stata module to perform full Mahalanobis and propensity score matching, common support graphing, and covariate imbalance testing. Published online 20030417. Accessed November 28, 2021. http://socionet.ru/publication.xml?h=repec:boc:bocode:S432001
